# Seasonal and Inter-Annual Variations of Carbon Dioxide Fluxes and Their Determinants in an Alpine Meadow

**DOI:** 10.3389/fpls.2022.894398

**Published:** 2022-06-23

**Authors:** Song Wang, Weinan Chen, Zheng Fu, Zhaolei Li, Jinsong Wang, Jiaqiang Liao, Shuli Niu

**Affiliations:** ^1^Key Laboratory of Ecosystem Network Observation and Modeling, Institute of Geographic Sciences and Natural Research, Chinese Academy of Sciences, Beijing, China; ^2^College of Resources and Environment, University of Chinese Academy of Sciences, Beijing, China; ^3^Laboratoire des Sciences du Climat et de l'Environnement (LSCE), CEA-CNRS-UVSQ, UMR8212, Gif-sur-Yvette, France; ^4^College of Resources and Environment, and Academy of Agricultural Sciences, Southwest University, Chongqing, China

**Keywords:** carbon fluxes, seasonal variation, inter-annual variation, eddy covariance, alpine meadow

## Abstract

The alpine meadow is one of the most important ecosystems on the Qinghai-Tibet Plateau (QTP) due to its huge carbon storage and wide distribution. Evaluating the carbon fluxes in alpine meadow ecosystems is crucial to understand the dynamics of carbon storage in high-altitude areas. Here, we investigated the carbon fluxes at seasonal and inter-annual timescales based on 5 years of observations of eddy covariance fluxes in the Zoige alpine meadow on the eastern Tibetan Plateau. We found that the Zoige alpine meadow acted as a faint carbon source of 94.69 ± 86.44 g C m^−2^ y^−1^ during the observation periods with large seasonal and inter-annual variations (IAVs). At the seasonal scale, gross primary productivity (GPP) and ecosystem respiration (Re) were positively correlated with photosynthetic photon flux density (PPFD), average daily temperature (Ta), and vapor pressure (VPD) and had negative relationships with volumetric water content (VWC). Seasonal variations of net ecosystem carbon dioxide (CO_2_) exchange (NEE) were mostly explained by Ta, followed by PPFD, VPD, and VWC. The IAVs of GPP and Re were mainly attributable to the IAV of the maximum GPP rate (GPP_max_) and maximum Re rate (Re_max_), respectively, both of which increased with the percentage of *Cyperaceae* and decreased with the percentage of *Polygonaceae* changes across years. The IAV of NEE was well explained by the anomalies of the maximum CO_2_ release rate (MCR). These results indicated that the annual net CO_2_ exchange in the alpine meadow ecosystem was controlled mainly by the maximum C release rates. Therefore, a better understanding of physiological response to various environmental factors at peak C uptake and release seasons will largely improve the predictions of GPP, Re, and NEE in the context of global change.

## Introduction

There are great uncertainties in estimating the carbon dioxide (CO_2_) budget of terrestrial ecosystems due to the inadequacies in the observational data and the incomplete conceptual framework (Hawkins and Sutton, 2009; Ito, 2019). Hence, understanding the dynamics of ecosystem carbon fluxes on different time scales and their control mechanisms is of great significance for accurately simulating and predicting terrestrial ecosystem carbon balances (Jia et al., 2016; Green et al., 2019).

At the seasonal scale, water availability and thermal conditions were considered to affect the dynamics of ecosystem carbon fluxes (Zhang et al., 2018; Li et al., 2019b). For example, in arid and semiarid ecosystems, the amount and distribution of precipitation have been shown to dominate seasonal ecosystem carbon fluxes (Jia et al., 2016; Hao et al., 2018). In contrast, many studies in cold regions found that thermal conditions were the main drivers of the carbon fluxes at the seasonal scale (Fu et al., 2009; Saito et al., 2009). At the annual scale, the temperature fluctuations and water availability have been reported as the most important climate factors in controlling the inter-annual variation (IAV) of the gross primary productivity (GPP), ecosystem respiration (Re), and net ecosystem CO_2_ exchange (NEE) at the global scale (Jung et al., 2017; Marcolla et al., 2017; Fernandez-Martinez et al., 2019). Compared to environmental factors, the impact of the biotic mechanisms underlying the IAV of ecosystem CO_2_ fluxes has been less explored. Recently, it has been proposed that the maximum daily net ecosystem productivity (NEP) during the CO_2_ uptake period (CUP; NEP_max_) dominated the IAV of NEE at the global scale (Fu et al., 2019), while the summer peak of GPP (GPP_max_) contributed more to the IAV of GPP than the photosynthetic phenology across North America (Xia et al., 2015). This indicates that community properties related to the maximum C uptake rate are crucial in determining annual C uptakes. However, the controlling factor of CO_2_ fluxes may be divergent among different climate and vegetation types. For instance, temperature determines CO_2_ fluxes in tropical ecosystems (Wang et al., 2014), but precipitation regulates the annual CO_2_ flux of semiarid ecosystems (Poulter et al., 2014), and the soil moisture and species composition have been found to interactively determine CO_2_ fluxes in dry meadows (Luan et al., 2016). Thus, the mechanism underlying the seasonal and IAV of ecosystem CO_2_ fluxes in those less studied regions still needs further investigation.

The alpine meadow ecosystem is one of the most important ecosystems on the Qinghai-Tibet Plateau (QTP), covering an area of ≈70 × 10^4^ km^2^ and accounting for ≈35% of QTP (Ni, 2002; Niu et al., 2017a). It stores about 17.6 Gt carbon, accounting for about 48% of QTP carbon storage (Wang and Zhou, 1999; Lv, 2006). A large amount of carbon was stored in the alpine meadow ecosystem due to the low temperature, high humidity, low soil humus decomposition rate, and high accumulation rate of organic matter (Saito et al., 2009). However, the alpine area is increasingly impacted by climate change with rising temperature and precipitation (Li et al., 2010, 2019a). Meanwhile, the alpine meadow ecosystem is highly susceptible to environmental changes (Liu and Chen, 2000; Wang et al., 2000; Cheng and Wu, 2007; Xu and Liu, 2007). The temperature in the alpine meadow ecosystem increases (0.3–0.4°C per decade) two times faster than the global average (Chen et al., 2013), and the temperature increases more significantly with the increase in altitude (Liu and Chen, 2000). Therefore, studying the carbon fluxes and their response to climate change in the alpine meadow ecosystem is imperative. A few studies about carbon fluxes over alpine meadow ecosystems have been conducted on the QTP. For example, Hao et al. (2011) and Wang et al. (2016) reported that these alpine meadow ecosystems were a weak net CO_2_ sink, but the carbon source or sink dynamic has great variations due to the changes in environmental factors. Under the background of increasing air temperature and precipitation (Li et al., 2010, 2019a; Chen et al., 2013), there will be more uncertainty in predicting carbon fluxes in alpine meadow ecosystems in the future. Hence, it is vital to explore the temporal variations of carbon fluxes and their drivers in alpine meadow ecosystems.

Eddy covariance technology provides a reliable approach to measuring the CO_2_ fluxes. This approach can measure NEE with precision, contributes to identify the characteristics of source/sink activities of various global ecosystems, and has been widely used to interpret whole-system variability (Braswell et al., 2005; Chen et al., 2019b; Peng et al., 2021). This study focuses on the carbon fluxes dynamic at seasonal and inter-annual timescales based on 5 years (2015–2018, 2020) of eddy covariance flux observation in Zoige alpine meadow on the eastern QTP. The specific objectives of this study are to (1) quantify CO_2_ dynamics at seasonal and inter-annual timescales for the Zoige alpine meadow; (2) understand the abiotic and biotic controlling factors for the variations in ecosystem CO_2_ fluxes; and (3) explore the key processes associated with plant community species in controlling the inter-annual variability of CO_2_ flux. These controlling mechanisms are essential to help us better understand the response of alpine meadows to future climate change.

## Materials and Methods

### Site Description

**Figure 1:**
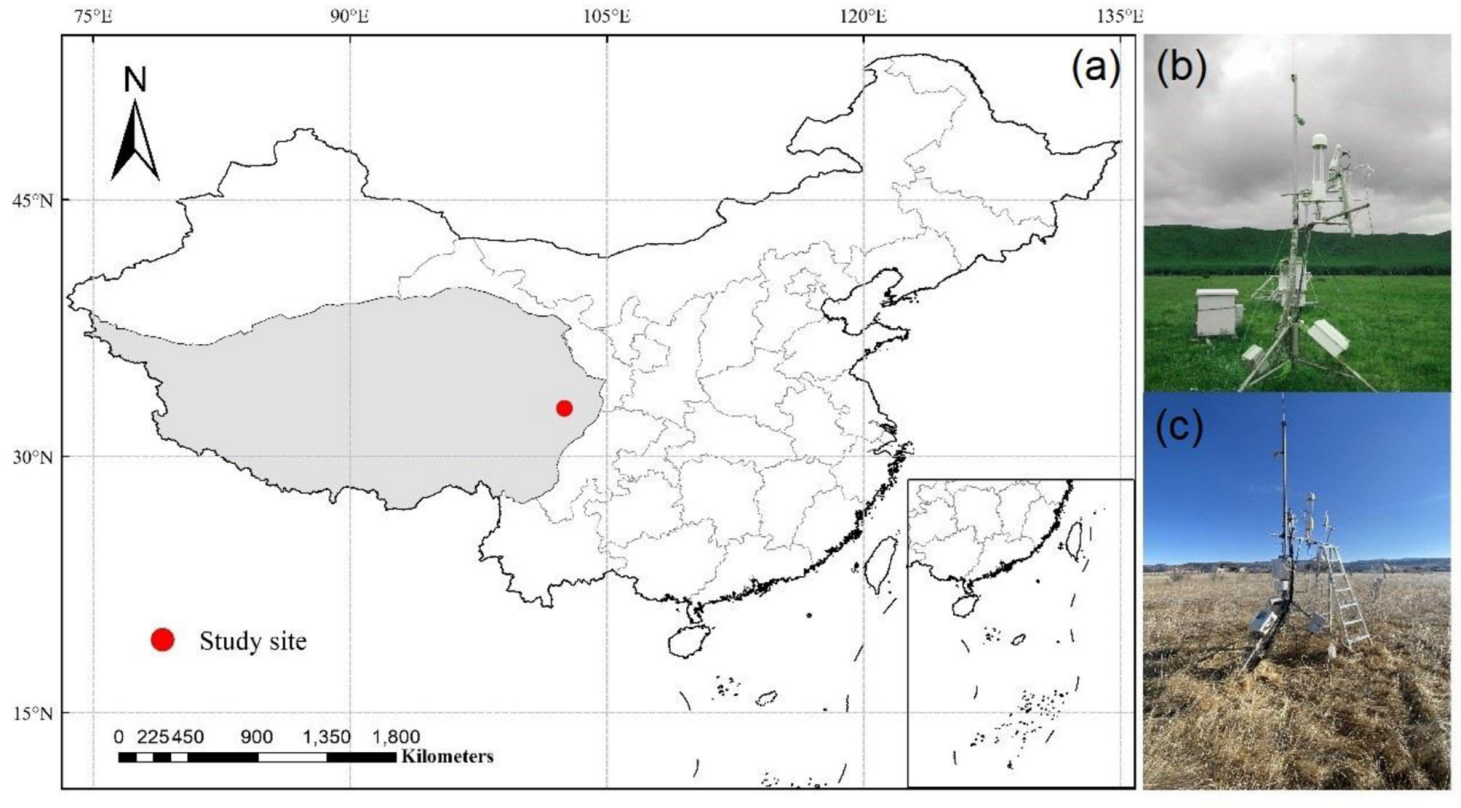
**(a)** Study site on the Tibetan Plateau and the eddy covariance measurements during the growing season, **(b)** and non-growing season, **(c)** at our study site.

The study site is at an alpine meadow in the National Zoige Alpine Wetland Ecological Station (32.8°N and 102.6°E; 3,500 m a.s.l), located on the eastern Qinghai-Tibetan Plateau (Figure 1). The alpine meadow is characterized by a typical continental plateau monsoon climate with relatively low temperatures and strong solar radiation. Based on the long-term meteorology observation data (1961–2013) from Hongyuan meteorological station (http://101.201.172.75:8888), the annual mean temperature of this site is ≈1.5°C. The coldest month occurs in January with a mean temperature of −9.7°C, while the warmest month occurs in July with a mean temperature of 11.1°C. The mean annual precipitation of this site is ≈761.0 mm, and over 80% of which occurs in the growing season (May to October).

The vegetation at the study site consists of a species mixture of *Deschampsia cespitosa* (Linn.) *Beauv., Koeleria cristata* (Linn.) Pers., *Gentiana sino-ornata* Balf. f., *Potentilla anserina* L., and *Anemone rivularis* Buch.-Ham (Quan et al., 2018). The dominant soil type in this ecosystem is Mat Cry-gelic Cambisol.

### Eddy Covariance and Meteorological Measurement

Net ecosystem CO_2_ exchange was observed from 2015 to 2020 by an open-path eddy covariance measurement system installed above an alpine meadow at 2 m. The sensor was broken at the beginning of 2019, so there was a long data gap in 2019 and the data in 2019 were discarded. The open-path eddy covariance system has a three-dimensional sonic anemometer (CSAT3; Campbell Scientific Inc. (CSI), Logan, USA) and an open-path CO_2_/H_2_O infrared gas analyzer (LI-7500A; Li-COR Inc, Lincoln, NE, USA). Flux data are logged with a data logger at 10 Hz (CR5000, Campbell Scientific, UT, USA). HMP45C temperature probe (Vaisala, Finland) was used to measure air temperature. Soil volumetric water content (VWC) at a depth of 5 and 10 cm was measured using a CS655 probe (CSI, Logan, USA). Precipitation was measured by a tipping bucket rain gauge (TE525, CSI, Logan, USA). Photosynthetic photon flux density (PPFD) was measured using a photosynthetic active radiation sensor (LI190, LI-Cor, USA). This eddy covariance tower is one of the ChinaFlux (China Flux Observation and Research Network) and FLUXNET long-term observation site.

### Aboveground Biomass Measurement

At the peak of annual biomass (usually in August), we randomly placed a quadrat frame (0.50) on each plot × 0.50 m), all the aboveground parts of the plants in the frame together, then separated them into different living species, and dried in the oven at 65°C for 48 h until they reached a constant weight and weighed. In the five replicates of each treatment, the average biomass of all living species in each quadrat was used to calculate aboveground biomass (Ma et al., 2020).

### Data Processing

EddyPro 6.2.0 software was used to preprocess and control the quality of the eddy covariance raw data. Data measured during instrument malfunction and severe conditions were filtered out. Specifically, for data quality control, half-hour CO_2_ flux data were filtered when: (1) data values were beyond the range of −20 to 20 μmol/m^2^/s; (2) precipitation occurred; and (3) the friction velocity (u^*^) was below 0.1 m/s at nighttime. This u^*^ threshold was determined following Reichstein et al. (2005). The positive values represent CO_2_ emission from the underlying surface to the atmosphere, while the negative values represent CO_2_ consumption from the atmosphere to soil (plants). Here, we divided CO_2_ flux data into two periods: (1) the growing season was between the day with daily mean Tair > 5 and the day with daily mean Tair <5°C for 7 consecutive days, (2) the non-growing season was the days of the year except the growing season (Lund et al., 2010; Song et al., 2015; Peng et al., 2021). GPP and Re data were partitioned from CO_2_ flux data (i.e., NEE) using rectangular hyperbolic regression (Falge et al., 2001). More information about missing NEE data gap-filling and partitioning was previously described by Chen et al. (2019b).

### Statistical Analysis

**Figure 2:**
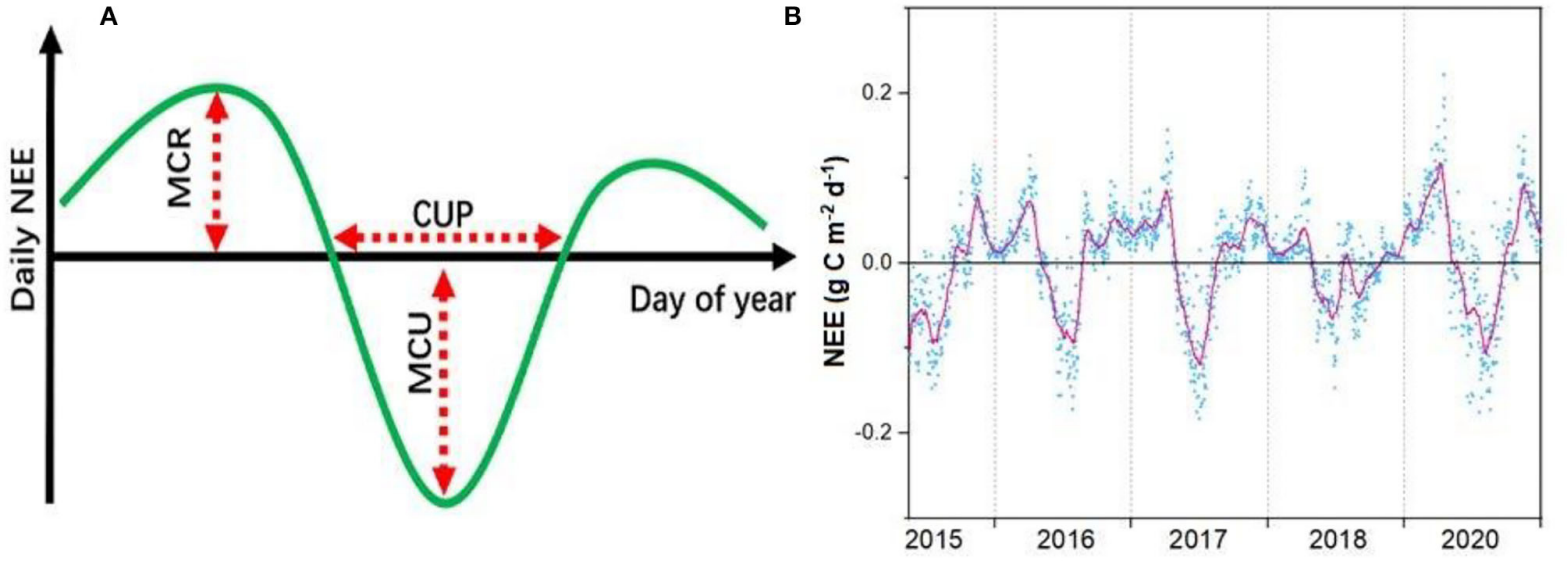
Conceptual figure of maximum CO_2_ uptake (MCU), CO_2_ uptake period (CUP), and maximum CO_2_ release (MCR) in determining the changes in annual net ecosystem CO_2_ exchange (NEE) **(A)** with examples of the annual course of observed and filtered NEE **(B)**.

We used the daily NEE to calculate the maximum CO_2_ uptake rate (MCU), net CUP, and maximum CO_2_ release rate (MCR) to quantify the phenological and physiological indicators that determine the annual NEE (Fu et al., 2019) and applied the Savitzky–Golay filter to minimize the role of random variability in flux observations (Figure 2) (Savitzky and Golay, 1964). We defined the CUP as the number of days with net C uptake (NEE <0 g C m^−2^ day^−1^) (Fu et al., 2019). Following this definition, there may be multiple periods across the course of a calendar year that may have net C uptake; these were added for the calculation of CUP on an annual basis. The MCU was defined as the maximum daily net C uptake of the filtered time series. The MCR was defined as the maximum value of the daily net C release of the filtered time series (Fu et al., 2019). To explore the underlying mechanism controlling annual CO_2_ exchanges, we split the annual NEE into growing season NEE (NEE_g_) and non-growing season NEE (NEE_ng_). We used the same method as those in Gu et al. (2009) to quantify the canopy photosynthetic phenology and fitted a 9-parameter Weibull function to the data to obtain the GPP_max_ and Re_max_ value of each year.

## Results

### Environmental Factors

**Figure 3:**
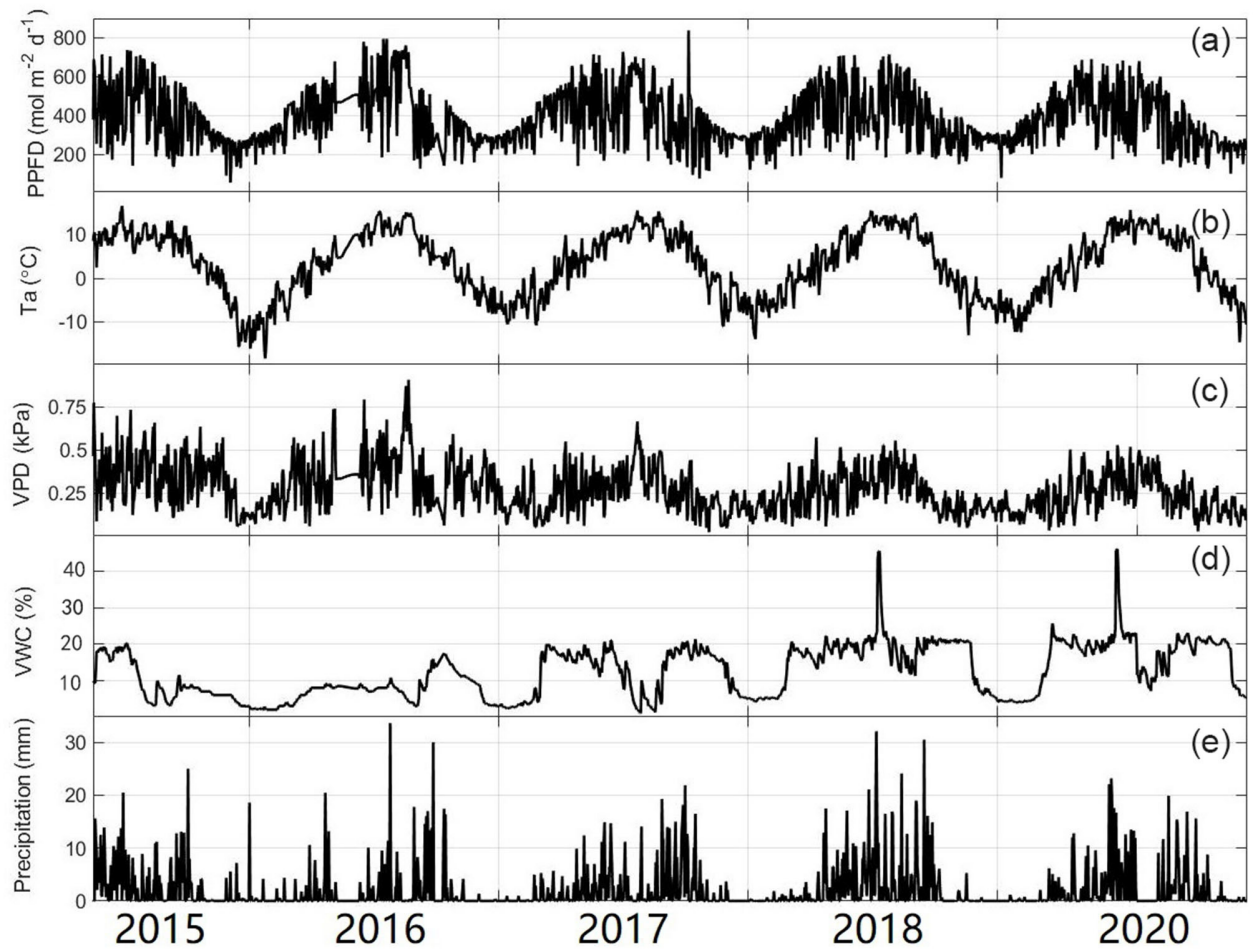
Seasonal variability of **(a)** photosynthetic photon flux density (PPFD), **(b)** average daily temperature (Ta), **(c)** vapor pressure (VPD), **(d)** volumetric water content (VWC), and **(e)** daily total precipitation. The lines are plotted from 1 January 1 to 31 December.

The daily mean Ta showed large seasonal variation, ranging from −18.4 to 15.67°C (Figure 3). The average annual temperature in this site from 2016 to 2020 was 0.44°C, of which 2020 was the coldest (0.15°C) year and 2017 was the warmest (0.66°C) year (Figure 3 and Table 1). Similar to temperature, PPFP showed a single peak in late June to early July each year. The maximum daily value could exceed 800 μmol photons m^−2^ d^−1^ (Figure 3). There were significant seasonal differences in daily precipitation, and the annual total precipitation amounts were 710.40, 860.80, 995.60, and 1,032.50 mm for 2016, 2017, 2018, and 2020, respectively (Table 1). The variation in the soil water content (SWC) and the mean vapor pressure deficit (VPD) was closely related to the precipitation at the study site. In addition, there were two sharp peaks in the VWC dynamic in 2018 and 2020 because our study site suffered flooding at that time.

**Table 1:** Climatic factors and carbon fluxes for 2016–2018 and 2020.

	**Year**					
	**2016**	**2017**	**2018**	**2020**	**Average**	**SD**
PPFD (mol m^−2^ d^−1^)	366.89	363.86	357.78	333.87	355.60	14.97
Ta (°C)	0.51	0.66	0.45	0.15	0.44	0.21
VPD (kPa)	0.32	0.22	0.22	0.20	0.24	0.05
VWC (100%)	0.07	0.12	0.14	0.15	0.12	0.04
Rain (mm)	710.40	860.80	995.60	1,032.50	899.82	146.26
ER (g C m^−2^ year^−1^)	869.36	889.89	556.42	1,306.91	905.64	307.37
GPP (g C m^−2^ year^−1^)	777.40	740.41	582.77	1,143.24	810.96	237.06
NEE (g C m^−2^ year^−1^)	91.96	149.48	−26.35	163.67	94.69	86.44

### Seasonal Variations of GPP, Re, and NEE and Their Controlling Factors

**Figure 4:**
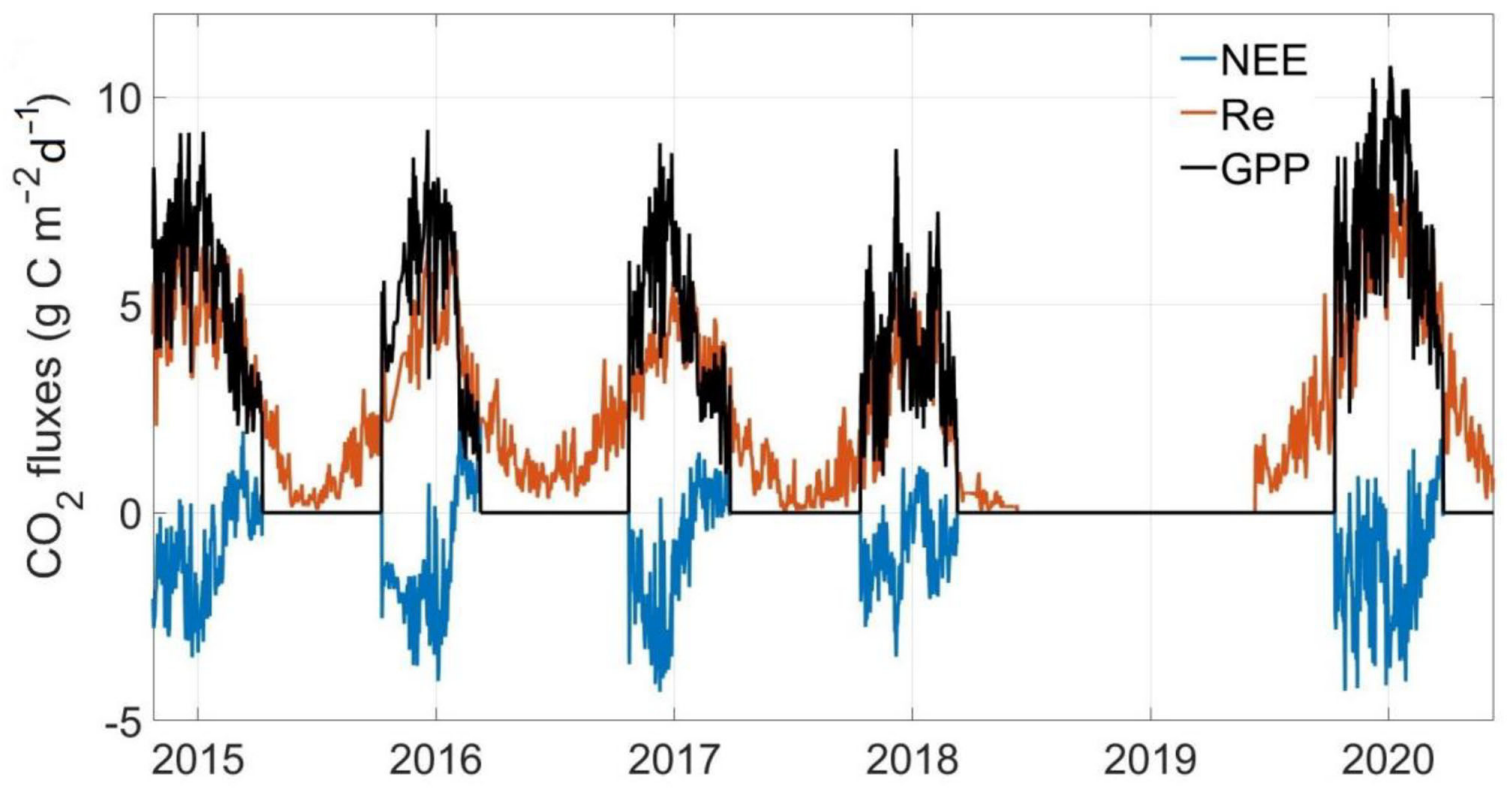
Seasonal and inter-annual variation in the daily average net ecosystem CO_2_ exchange (NEE), gross primary productivity (GPP), and ecosystem respiration (Re).

In all observational years, GPP and Re both showed similar curvilinear shapes, with zero GPP and very low Re in the non-growing season (Figure 4). The maximum daily GPP values were 4.74–8.60 g C m^−2^ day^−1^ among 4 research years. The daily Re was low in winter (<0.5 g C m^−2^ day^−1^) with the maximum values of 4.63–6.77 g C m^−2^ day^−1^ in 4 research years. The maximum daily NEE value in the growing season was about −2.39 g C m^−2^ day^−1^, and the maximum daily NEE value in the non-growing season was about 2.08 g C m^−2^ day^−1^.

**Figure 5:**
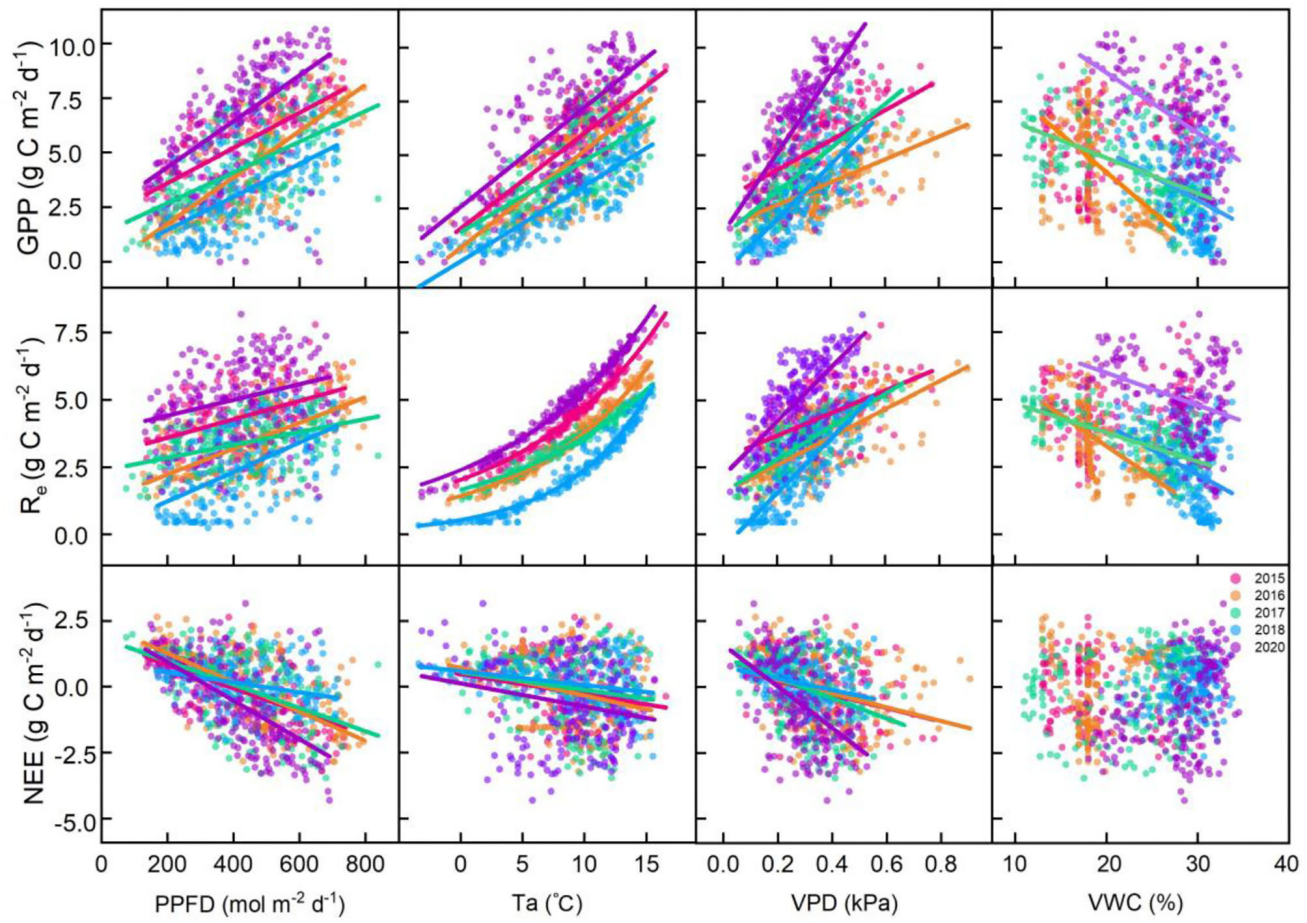
Relationships between daily carbon fluxes (gross primary productivity [GPP], ecosystem respiration [Re], and net ecosystem carbon dioxide (CO_2_) exchange [NEE]) and environmental factors (the photosynthetic photon flux density (PPFD), air temperature (Ta), vapor pressure deficit (VPD), and volumetric water content at a depth of 5 cm (VWC at 5 cm)). The fitted lines indicate that the regressions are significant under the confidence of 0.05.

At the seasonal scale, GPP and Re were positively correlated with PPFD, Ta, and VPD and negatively correlated with VWC at 5 cm. Re and Ta showed a significant exponential relationship (*p* < 0.001; Figure 5). NEE was negatively correlated with PPFD, Ta, and VPD and positively correlated with VWC (*p* < 0.01; Figure 5). We analyzed the relative contributions of six environmental variables to fluxes using the random forest (RF) scheme. The RF of this alpine meadow ecosystem explained 80.01 and 78.93% of the daily GPP and Re variations during the vegetative periods, respectively (Supplementary Table S1). Meanwhile, the RF explained 53.11% of the variations in the daily NEE (Supplementary Table S1).

### IVVs of GPP, Re, and NEE and Their Controlling Factors

In general, the yearly cumulative GPP was 810.96 ± 237.06 g C m^−2^, and the total accumulative Re was 905.64 ± 307.37 g C m^−2^ over 4 study years (Figure 4 and Table 1). The alpine meadow ecosystem was a faint carbon source during the observation period. The mean NEE of these 4 years was 94.69 ± 86.44 C m^−2^ year^−1^ although the NEE in 2018 was negative. The positive NEE values indicated a net emission of CO_2_ in the alpine meadow ecosystem during these 4 years.

**Figure 6:**
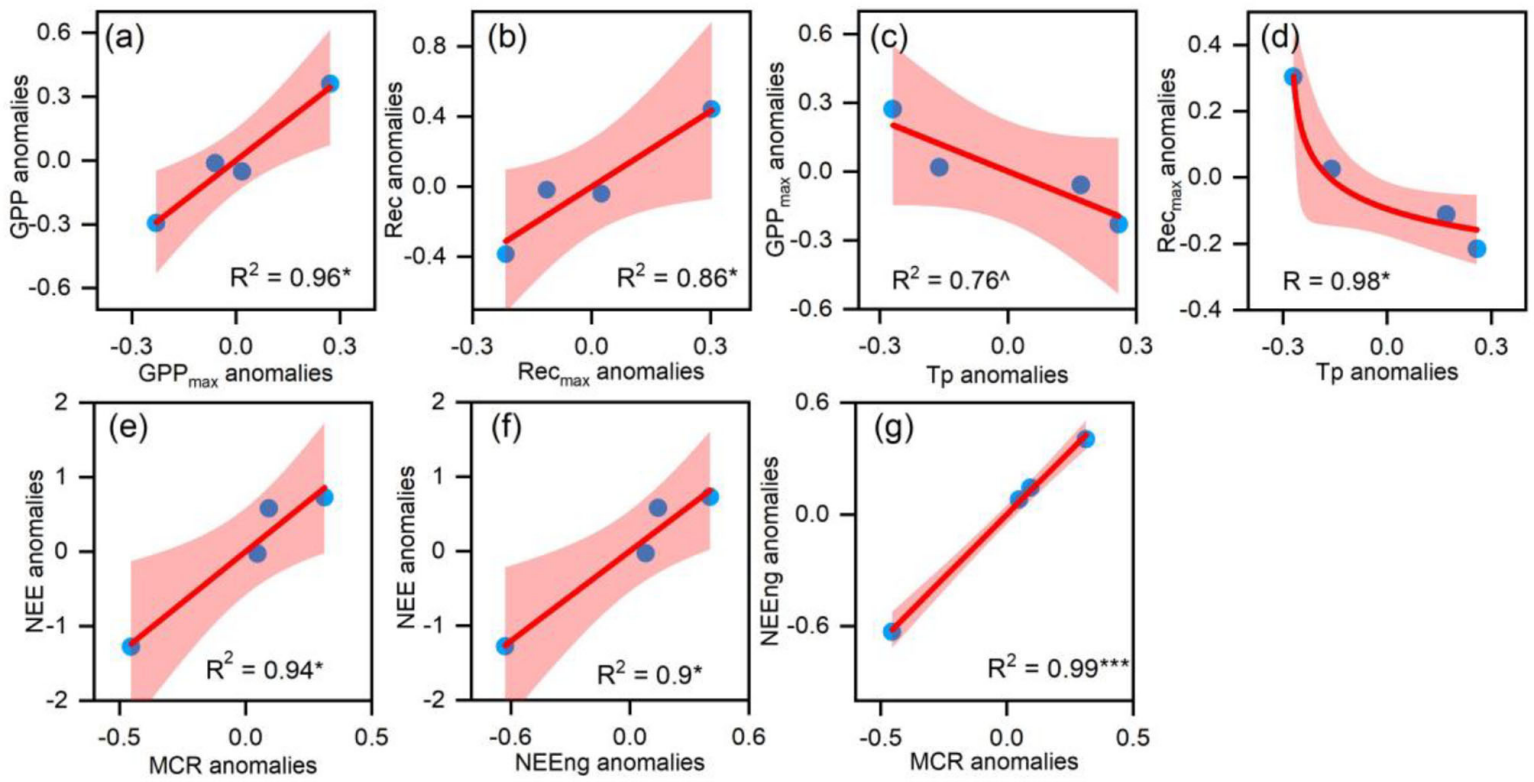
**(a)** The relationship between the yearly gross primary productivity (GPP) anomalies and the GPP_max_ anomalies; **(b)** the relationship between the yearly Re anomalies and the Re_max_ anomalies; **(c,d)** the relationship between the GPP_max_ anomalies, the Re_max_ anomalies, and the preseason temperature (Tp) anomalies and; **(e–g)** the relationship between the MCR anomalies, the NEE_ng_ anomalies, and the NEE anomalies. ^∧^when *p* < 0.1, * when *p* < 0.05, ** when *p* < 0.01, ***when *p* < 0.001.

There were no significant correlations between environmental factors and CO_2_ fluxes on the annual scale. The yearly GPP anomalies and Re anomalies were significantly related to GPP_max_ animalizes (Figure 6a) and Re_max_ anomalies, respectively (Figure 6b). Moreover, the anomalies of GPP_max_ and Re_max_ were negatively correlated with the preseason temperature (Tp, the average temperature from February to April) (Figures 6c,d). NEE anomalies were positively correlated with the IAV of MCR (Figure 6e) but had no significant corrections with MCU or CUP. Meanwhile, annual NEE anomalies were positively correlated with the IAV of accumulated NEE in the non-growing season (Figure 6f), which were significantly related to MCR anomalies (Figure 6g).

**Figure 7:**
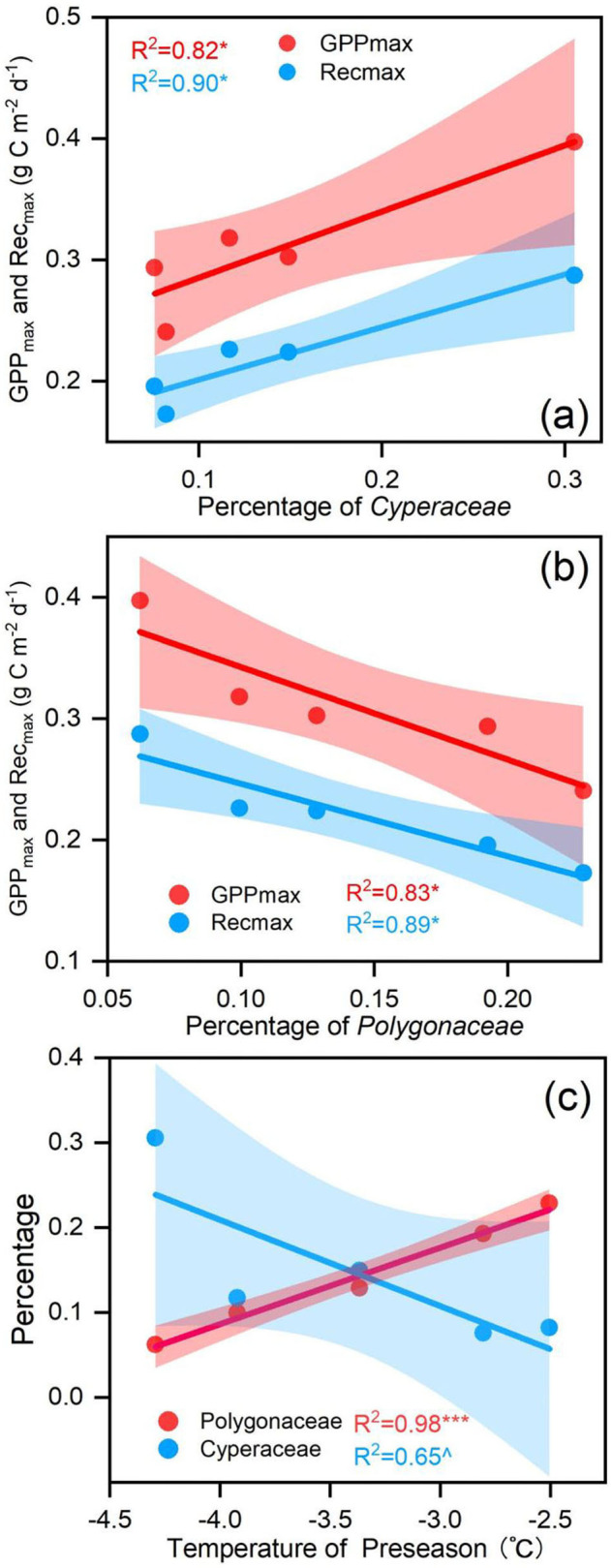
**(a)** the relationship between the percentage of *Polygonaceae* and carbon fluxes; **(b)** the relationship between the percentage of *Cyperaceae* and carbon fluxes; **(c)** the relationship between the percentage of *Polygonaceae, Cyperaceae* and the temperature of the preseason (Tp). ^∧^when *p* < 0.1, * when *p* < 0.05, ** when *p* < 0.01, ***when *p* < 0.001.

Biological factors were also considered in this study. Plant community varied considerably during the observation period (Supplementary Figure S1). The percentage of *Cyperaceae* had a positive relationship with the GPP_max_ and Re_max_ (Figure 7a). The percentage of *Polygonaceae* had a negative relationship with the GPP_max_ and Re_max_ (Figure 7b). In addition, the higher Tp had a negative effect on the percentage of *Cyperaceae* (Figure 7c) but had a positive effect on the percentage of *Polygonaceae* (Figure 7c) on the annual scale.

## Discussion

### CO_2_ Budget at Zoige Alpine Meadow

The carbon budget at Zoige alpine meadow in this study was not consistent with most alpine meadow ecosystems in the Qinghai-Tibet region, which usually acted as a carbon sink (Kato et al., 2006; Zhao et al., 2006; Sun et al., 2019; Wang et al., 2020). Different from the previous opinion that the favorable photosynthetic conditions and a low decomposition rate of organic matter result in carbon accumulation in alpine meadow ecosystems (Kato et al., 2006; Fu et al., 2009), the net carbon balance performed as a weak source among these 4 years in this study. Because the GPP and Re values were comparable in the growing season, the carbon accumulation during the growing season was less than the respiration in the non-growing season.

In this study, both the GPP (810.96 g C m^−2^ year^−1^) and Re (905.64 g C m^−2^ year^−1^) values were very high in comparison with other alpine meadow ecosystems (Tables 1, 2). The relatively high precipitation and temperature led to higher productivity and greater respiration consumption. However, the high productivity did not lead to net carbon uptake accumulation during the observation period because the Re in the cold ecosystem was large and more sensitive to the environmental change than GPP (Illeris et al., 2004; Zhu et al., 2016). For instance, favorable weather increased the Re and GPP, leading to a net carbon emission of 163.67 g C m^−2^ in 2020. However, unfavorable weather decreased the Re and GPP, leading to a net carbon sink of 26.35 g C m^−2^ in 2018.

**Table 2:** Ecosystem carbon fluxes in other alpine meadows published in previous studies.

**Type**	**Site**	**Period**	**Ta** **°C**	**GPP** g C m^**−2**^ y^**−1**^	**Re** g C m^**−2**^ y^**−1**^	**NEE** g C m^**−2**^ y^**−1**^	**Reference**
Alpine meadow	Haibei	2003–2004	−1.48	—	—	−282	Zhao et al. (2005)
Alpine shrub meadow				—	—	−53	
Alpine meadows				—	—	478	
Alpine shrub-meadow	Haibei	2004–2005	−1.7	527.7	459.2	−68.5	Fu et al. (2009)
Alpine meadow- steppe	Dangxung		1.3	205.8	253.8	48	
Alpine steppe	Bange	2015	0.02	—	—	21.8	Wang et al. (2018)
Alpine meadow	Lijiang		6.16	—	—	−230	
Alpine meadow	Arou	2013–2016	0.6	818.3	619.6	−198.7	Sun et al. (2019)
Alpine meadow	Dashalong		−3.4	467.5	208.6	−258.9	
Alpine meadow	Yakou	2015–2016	−4.2	228.6	123.3	−105.3	
Alpine wetland	Luanhaizi	2007–2016	−1.1	500.3	620.7	120.4	Zhu et al. (2020)
Alpine meadow	Haibei	2002–2004	−1	634.5	513.6	−120.9	Kato et al. (2006)
Alpine wetland meadow	Haibei	2004–2006	−1.1	629.9	737.1	107.2	Zhao et al. (2010)
Alpine shrubland meadow	Haibei	2003–2004		551.7	484.6	−67.1	Zhao et al. (2006)
Alpine meadow	Hongyuan	2015–2020	0.44	810.96	905.64	94.69	This study

### Environmental Controls on Seasonal Variation of Ecosystem CO_2_ Fluxes

Previous studies have shown that carbon fluxes had a clear seasonal dynamic in temperate and cold ecosystems (Zhao et al., 2010; Niu et al., 2017a; Wang et al., 2020). Alpine meadow ecosystem had a low-temperature condition, and the temperature and thermal conditions were often the limiting factors for vegetation growth, which was typically considered the main factor regulating carbon fluxes (Saito et al., 2009; Li et al., 2019b). The RF analysis suggested that Ta primarily influenced the seasonal changes in GPP, Re, and NEE in this alpine meadow ecosystem, and SWC played subordinate roles in affecting seasonal GPP, Re, and NEE changes (Supplementary Table S1).

This result was consistent with previous studies, which found that temperature was the most critical factor for controlling NEE, GPP, and Re for an alpine meadow ecosystem (Kato et al., 2006; Fu et al., 2009; Saito et al., 2009). The reason was that although cold and humid environments provided an adequate soil water supply for plants growth during the growing season, the low temperature often became a limiting factor for plant growth, as temperature affected both the physiology and phenology of plants, which in turn determined the carbon uptake and release. In addition to Ta, SWC played a subordinate role in affecting seasonal GPP, Re, and NEE changes. Previous studies had shown that soil moisture had an important effect on controlling carbon fluxes for a water-limited ecosystem (Wang et al., 2008; Ganjurjav et al., 2016; Zhang et al., 2018) but had little effect on water-rich areas (i.e., wetland) (Zhao et al., 2010; Du et al., 2021). The soil water supply in the Zoige alpine meadow ecosystem was intermediate between grassland and wetland, leading to the subordinate role in seasonal carbon flux variations.

### Controlling Factors on IAV in GPP and Re

Our study demonstrated that the IAV of GPP_max_ and Re_max_ mostly determined the IAV of GPP and Re in Zoige alpine meadow, respectively, instead of plant phenology or climates. This result was consistent with previous studies that found the IAV of GPP was best explained by that in GPP_max_ in North America, Europe, and the Tibetan Plateau (Xia et al., 2015; Zhou et al., 2016; Chen et al., 2019a), and the IAV of Re was mainly attributed to Re_max_ at Maoershan forest (Liu et al., 2021a). The control of GPP_max_ on annual GPP variability and the Re_max_ on annual Re variability indicated that environmental changes influenced the IAVs of GPP and Re by affecting vegetation physiology rather than phenology. Hence, given GPP_max_ and Re_max_'s importance for the alpine region's carbon cycle, it was vital to explore the physiological mechanism underlying GPP_max_ and Re_max_ change in the alpine ecosystem.

The maximum GPP rate was determined by the leaf area index and the leaf photosynthetic capacity of the ecosystem (Hu et al., 2018), and the Re_max_ was also tightly associated with plant biomass and vegetable characteristics (e.g., temperature sensitivity) (Kato et al., 2004a,b; Flanagan and Johnson, 2005; Yashiro et al., 2010). These physiological factors were greatly affected by the change in plant community structure (Johnson et al., 2008; Cheng et al., 2015; Xu et al., 2015; Estruch et al., 2018). It had been widely reported that the species composition in the alpine meadow was shifting due to destruction by rodents (Zhou et al., 2005), climate change (Li et al., 2011), or other uncertain causes (Harris, 2010). These interferences could affect ecosystem processes through changing plant species composition (Poulter et al., 2014) and thus ecosystem functions (i.e., GPP and Re) (Sala et al., 2012; Kulmatiski and Beard, 2013).

Our observed changes in species' community support the above explanation. During the study period, the species communities changed significantly from a *Poaceae*-dominated meadow in 2015 to a *Cyperaceae*-dominated meadow in 2020 (Supplementary Figure S1). In our study site, *Cyperaceae, Polygonaceae, Euphorbiaceae, Poaceae*, and *Asteraceae* accounted for more than 80% of aboveground biomass (Supplementary Figure S1). A previous study had shown that *Cyperaceae* and *Poaceae* had the highest photosynthetic rate and water use efficiency among all of the function groups, and the photosynthetic rate of *Polygonaceae* was the lowest (Liu et al., 2015). Meanwhile, a study based on isotope labeling also found that *Cyperaceae* plants have a stronger ability to assimilate CO_2_ and transfer more C to roots and soil, because *Cyperaceae* plants had a high primary carbon assimilation tissue area when compared with *Poaceae* (Mou et al., 2018).

Hence, the percentage of *Cyperaceae* and *Polygonaceae* explained the annual GPP_max_ and Re_max_ variations in Zoige alpine meadow ecosystem. A greater proportion of *Cyperaceae* and a smaller proportion of *Polygonaceae* contributed to a larger annual GPP_max_. Meanwhile, we found that the temperature before the growing reason had a negative effect on the percentage of *Cyperaceae* (*p* < 0.1; Figure 7c) but had a positive effect on the percentage of *Polygonaceae* (*p* < 0.01; Figure 7c) on the annual scale. Consequently, a warmer preseason could reduce annual GPP_max_ by increasing the percentage of *Polygonaceae* and decreasing the percentage of *Cyperaceae*.

### Controlling Factors on IAV in NEE

Any single environmental factor could not explain the IAV of NEE in this study. Instead, it was explained well by biological processes, such as NEE_ng_ and MCR. The environmental driving factors may ultimately impact the IAV of NEE by changing the phenological and physiological indicators (Fu et al., 2017; Niu et al., 2017b).

Moreover, we found that the IAV of NEE at Zoige alpine meadow was primarily explained by the physiological (MCR) rather than phenological indicators (CUP). Surprisingly, the MCU did not affect the IAV of NEE, which indicated that the IAV of NEE was driven mainly by the net CO_2_ release process during the non-growing season rather than the net CO_2_ uptake during the growing season in this area, although NEE in growing and non-growing seasons was determined predominately by MCU and MCR, respectively. Meanwhile, the CUP tended to have no significant influence on either NEE_g_ or NEE_ng_. A global study also indicated that the CUP contribution to IAV of NEE was lower than the physiological indicator in Zoige alpine meadow area (Fu et al., 2019). Moreover, a study conducted on Siberian tundra also showed that the IAV of NEE had no significant relationship with CUP because of the offset effect between GPP and Re (Parmentier et al., 2011).

However, the driving factor of the IAV of NEE in this study differed from some other studies that indicated the IAV of NEE was determined predominately by MCU (Zscheischler et al., 2016; Gonsamo et al., 2018; Fu et al., 2019). Because most of the study sites in these research studies were carbon sinks, carbon uptake in the growing season was larger than the carbon release in the non-growing season, resulting in the dominant role of MCU in contributing to the IAV of NEE. In our study site, the mean NEE over the 4 years was 94.69 ± 86.44 C m^−2^ year^−1^. The carbon uptake in the growing season was smaller than the carbon release in the non-growing season, leading to the dominant role of MCR in contributing to the IAV of NEE in this study.

We were aware of the possible uncertainty of IAV in GPP and Re due to the short observation periods in this study. The controlling mechanisms for the IAV of GPP and Re could be different in short-term and long-term series because the effects from the influencing factors were changing over time (e.g., legacy effects and accumulation effects) (Bloom et al., 2020; Liu et al., 2021b), and the ecosystems were also acclimating to the changing environments (Luo et al., 2001; Guo et al., 2020). Hence, we suggest that more research should be conducted to explore the processes that control the long-term IAV of GPP, Re, and NEE in the future.

## Conclusion

The Zoige alpine meadow acted as a faint carbon source during the observation period. GPP, Re, and NEE all showed strongly seasonal and IVVs. The seasonal variations of GPP, Re, and NEE were mostly determined by Ta, followed by PPFD, VPD, and VWC, while GPP_max_ and Re_max_ drove the IAV of GPP and Re. Meanwhile, the higher Tp could decrease the GPP_max_ and Re_max_ by changing the plant species composition in the growing season and decrease GPP and Re in Zoige alpine meadow. The IAV of NEE at the Zoige alpine meadow was largely explained by the MCR, indicating the important role of carbon release in the non-growing season in determining the net C sink in the alpine region. Given the physiological indicators (i.e., GPP_max_, Re_max_, and MCR) can best explain the CO_2_ exchange variability, future studies need to emphasize the regulatory mechanisms for the dynamics of ecosystem physiological characteristics in the alpine ecosystem.

## Data Availability Statement

The raw data supporting the conclusions of this article will be made available by the authors, without undue reservation.

## Author Contributions

SW, WC, and SN designed this study. SW performed the laboratory analysis and wrote the paper. All authors have revised, discussed, and approved the final manuscript.

## Funding

This study was supported by the National Natural Science Foundation of China (31988102) and the Second Tibetan Plateau Scientific Expedition and Research (STEP) program (2019QZKK0302).

## Conflict of Interest

The authors declare that the research was conducted in the absence of any commercial or financial relationships that could be construed as a potential conflict of interest.

## Publisher's Note

All claims expressed in this article are solely those of the authors and do not necessarily represent those of their affiliated organizations, or those of the publisher, the editors and the reviewers. Any product that may be evaluated in this article, or claim that may be made by its manufacturer, is not guaranteed or endorsed by the publisher.
