# Supplementary material for: Seasonal and Inter-Annual Variations of Carbon Dioxide Fluxes and Their Determinants in an Alpine Meadow
Source: Front Plant Sci. 2022 Jun 23;13:894398. doi: 10.3389/fpls.2022.894398 (PMC9260316; doi:10.3389/fpls.2022.894398)
Supplement: Supplementary file 1 [file Table_1.docx]

*[Frontiers in Plant Sciences]*

Supplementary Material

**[Seasonal and inter-annual variations of carbon dioxide fluxes and their determinants in an alpine meadow]**

[Song Wang^1,2^, Weinan Chen^1,2^, Zheng Fu^3^, Zhaolei, Li^4^, Jinsong Wang^1^, Jiaqiang Liao^1,2^, Shuli Niu^1,2*^]

[*^1^Key Laboratory of Ecosystem Network Observation and Modeling, Institute of Geographic Sciences and Natural Research, Chinese Academy of Sciences, Beijing 100101, China.*

*^2^College of Resources and Environment, University of Chinese Academy of Sciences, Beijing 100049, China.*

*^3^Laboratoire des Sciences du Climat et de l’Environnement (LSCE), CEA-CNRS-UVSQ, UMR8212, 91191 Gif-sur-Yvette, France.*

*^4^College of Resources and Environment, and Academy of Agricultural Sciences, Southwest University, Chongqing, China*]

Table S1 Relative importance of environmental variables in determining the seasonl GPP, Re and NEE variations.

| %LncMSE | GPP | Re | NEE |
| --- | --- | --- | --- |
| Ta | 311 | 349 | 302 |
| VWC | 140 | 130 | 165 |
| VPD | 125 | 112 | 159 |
| PPFD | 107 | 105 | 138 |
| Precipitation | 62 | 53 | 61 |
| Total explanation | 80.01% | 78.93% | 53.11% |


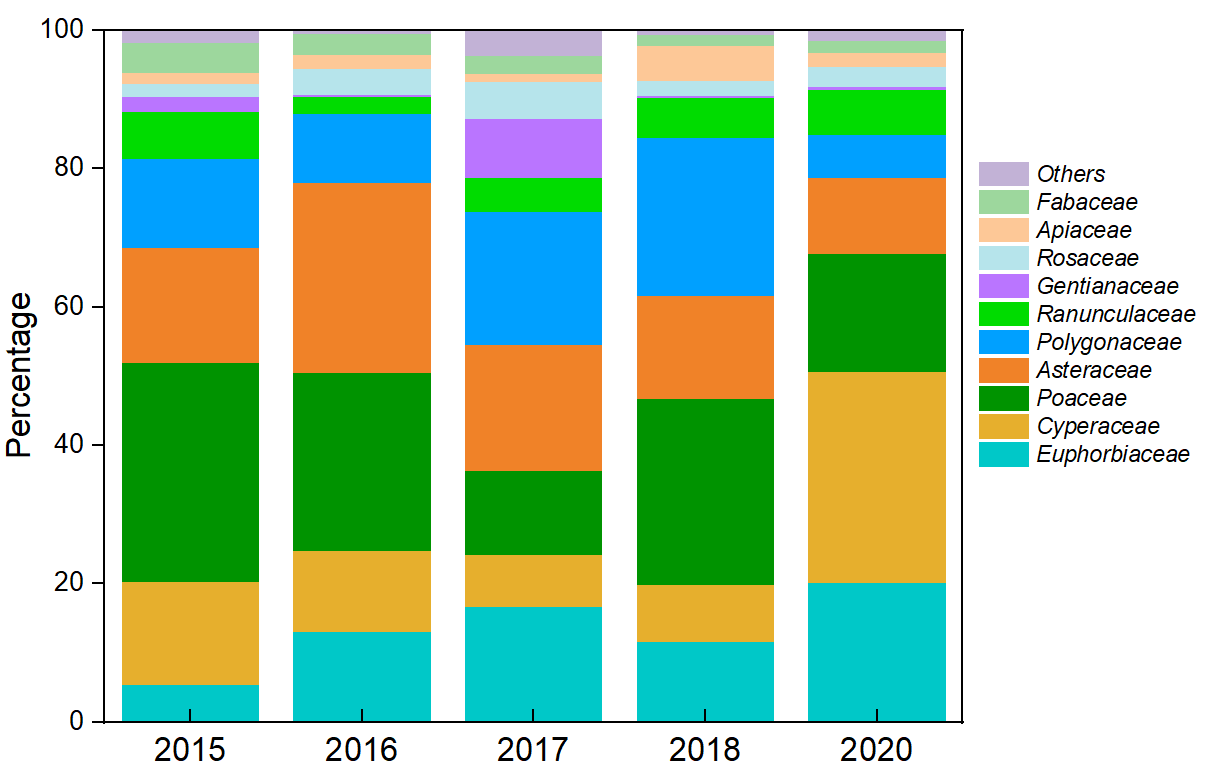


Figure S 1 the species compositions in this study during the observation period.
